# Prevalence, misclassification, and clinical consequences of the heteroresistant phenotype in *Escherichia coli* bloodstream infections in patients in Uppsala, Sweden: a retrospective cohort study

**DOI:** 10.1016/j.lanmic.2024.101010

**Published:** 2025-01-16

**Authors:** Gabriel Heyman, Sofia Jonsson, Nikos Fatsis-Kavalopoulos, Karin Hjort, Hervé Nicoloff, Mia Furebring, Dan I Andersson

**Affiliations:** Department of Infectious Diseases, Västmanland County Hospital Västerås, Västerås, Sweden (G Heyman MD); Centre for Clinical Research Västmanland, Västmanland County Hospital, Uppsala University, Västerås, Sweden (G Heyman); Department of Medical Biochemistry and Microbiology, Uppsala University, Uppsala, Sweden (S Jonsson MSc, N Fatsis-Kavalopoulos PhD, K Hjort PhD, H Nicoloff PhD, Prof D I Andersson PhD); Department of Infectious Diseases, Uppsala University Hospital, Uppsala, Sweden (M Furebring MD PhD)

## Abstract

**Background:**

Antibiotic heteroresistance is a common bacterial phenotype characterised by the presence of small resistant subpopulations within a susceptible population. During antibiotic exposure, these resistant subpopulations can be enriched and potentially lead to treatment failure. In this study, we examined the prevalence, misclassification, and clinical effect of heteroresistance in *Escherichia coli* bloodstream infections for the clinically important antibiotics cefotaxime, gentamicin, and piperacillin–tazobactam.

**Methods:**

We conducted a retrospective cohort analysis of patients (n=255) admitted to in-patient care and treated for *E coli* bloodstream infections within the Uppsala region in Sweden between Jan 1, 2014, and Dec 31, 2015. Patient inclusion criteria were admission to hospital on suspicion of infection, starting systemic antibiotics at the time of admission, positive blood cultures for the growth of *E coli* upon admission, and residency in the Uppsala health-care region at the time of admission. Exclusion criteria were growth of an additional pathogen than *E coli* in blood cultures taken at admission or previous inclusion of the patients in the study for another bloodstream infection. Antibiotic susceptibility of preserved blood culture isolates of *E coli* was assessed for cefotaxime, gentamicin, and piperacillin–tazobactam by disk diffusion and breakpoint crossing heteroresistance (BCHR) was identified using population analysis profiling. The clinical outcome parameters were obtained from patient records. The primary outcome variable was length of hospital stay due to the *E coli* bloodstream infection, defined as the time between admission and discharge from inpatient care as noted on the physician’s notes. Secondary outcomes were time to fever resolution, admission to intermediary care unit or intensive care unit during time in hospital, switching or adding another intravenous antibiotic treatment, re-admission to hospital within 30 days of original admission, recurrent *E coli* infection within 30 days of admission to hospital, and all-cause mortality within 90 days of admission.

**Findings:**

A total of 255 participants with a corresponding *E coli* isolate (out of 500 screened for eligibility) met the inclusion criteria, with 135 female patients and 120 male patients. One (<1%) of 255 strains was BCHR for cefotaxime, 109 (43%) of 255 strains were BCHR for gentamicin, and 22 (9%) of 255 strains were BCHR for piperacillin–tazobactam. Clinical susceptibility testing misclassified 120 (96%) of 125 heteroresistant bacterial strains as susceptible. The BCHR phenotypes had no correlation to length of hospital stay due to the *E coli* bloodstream infection. However, patients with piperacillin–tazobactam BCHR strains who received piperacillin–tazobactam had 3⋅1 times higher odds for admittance to the intermediate care unit (95% CI 1⋅1–9⋅6, p=0⋅041) than the remainder of the cohort, excluding those treated with gentamicin. Similarly, those infected with gentamicin BCHR who received gentamicin showed higher odds for admittance to the intensive care unit (5⋅6 [1⋅1–42⋅0, p=0⋅043]) and mortality (7⋅1 [1⋅2–49⋅2, p=0⋅030]) than patients treated with gentamicin who were infected with non-gentamicin BCHR *E coli.*

**Interpretation:**

In a cohort of patients with *E coli* bloodstream infections, heteroresistance is common and frequently misidentified in routine clinical testing. Several negative effects on patient outcomes are associated with heteroresistant strains.

**Funding:**

Wallenberg Foundation, Swedish Research Council, and US National Institutes Of Health.

## Introduction

Heteroresistance refers to a phenomenon in which a primarily susceptible bacterial population contains small subpopulations of antibiotic resistant cells.^1,2^ Heteroresistance is usually defined as the presence of resistant subpopulations at a frequency of at least 1×10^–7^ with a resistance at least eight times higher than the susceptible main population.^3,4^ When an antibiotic is applied during treatment these resistant subpopulations can grow to higher frequencies and potentially cause treatment failure.^4^ Although the enrichment of resistant subpopulations in the presence of antibiotics in vitro and in animal models is well documented, the clinical significance of heteroresistance remains unclear.^1,3,5,6^ Some retrospective studies have shown increased mortality or worse clinical outcome among patients infected with vancomycin-heteroresistant *Staphylococcus aureus* and coagulase-negative staphylococci.^7–10^

The dynamic nature of heteroresistance, characterised by rare and often unstable subpopulations, poses a challenge for regular phenotypic antibiotic susceptibility tests.^11^ The standard test for heteroresistance is the population analysis profile (PAP) test that establishes both the frequency and increase in resistance of the subpopulations. This method is laborious and costly,^3,12^ and therefore not commonly used in clinical settings. Using conventional antibiotic susceptibility testing such as disk diffusion there is a known risk of classifying heteroresistant isolates as susceptible due to the low frequency of the subpopulations.^11,13^

*Escherichia coli* is the most common cause of bloodstream infections, yet not much is known about heteroresistance in *E coli* and its clinical significance.^14,15^ Available studies focus on broader spectrum agents such as carbapenems for which heteroresistance has been found;^4,16^ however, cefotaxime and piperacillin–tazobactam, the most commonly used intravenous antibiotics for serious *E coli* infections in Sweden, are not well studied. Although heteroresistance against piperacillin–tazobactam has been shown to occur, its prevalence and effect on treatment outcome remains unknown.^17^ In this study, we examined the prevalence, misclassification, and clinical effect of heteroresistance in *E coli* bloodstream infections for the clinically important antibiotics cefotaxime, gentamicin, and piperacillin–tazobactam.

## Methods

### Study design and participants

We conducted a retrospective cohort study on preserved blood culture isolates of *E coli.* Identification of resistant subpopulations by PAP test was combined with clinical data from the respective patient’s medical records. The study population consisted of all patients admitted to inpatient care and treated for *E coli* bloodstream infections within the Uppsala region in Sweden between Jan 1, 2014, and Dec 31, 2015. Participants were identified using the lab information system at the Department of Clinical Microbiology in Uppsala. Patient inclusion criteria were admission to hospital on suspicion of infection, started on systemic antibiotics at the time of admission, had positive blood cultures for the growth of *E coli* upon admission, and residency in the Uppsala health-care region at the time of admission. Exclusion criteria was growth of an additional pathogen than *E coli* in blood cultures taken at admission. A patient could only be included once, even if they suffered several bloodstream infections during the study period. This study was approved by the ethics board of the Swedish Ethical Review Authority (2020–00516).

### Procedures

In the analysis of the clinical effect of heteroresistance, the exposure was antibiotic treatment to which the infecting *E coli* isolate is known to harbour heteroresistance as determined by PAP tests. The primary outcome was length of hospital stay due to the *E coli* bloodstream infection, defined as the time between admission and discharge from in-patient care as noted on the physician’s notes. Secondary outcomes were time to fever resolution, admission to intermediary care unit (IMCU) or intensive care unit (ICU) during time in hospital, switching or adding another intravenous antibiotic treatment (ie, treatment failure), re-admission to hospital within 30 days of original admission, recurrent *E coli* infection within 30 days of admission to hospital, and all-cause mortality within 90 days of admission to hospital.

The following clinical data were obtained for each participant from electronical health records: sex and age at the time of admission, underlying conditions and Charlson Comorbidity Index (CCI) at the time of admission, previous antibiotic exposure during 90 days before admission to hospital, vital parameters (pulse rate, systolic blood pressure, respiratory rate, oxygen saturation, mental status and body temperature) and National Early Warning Score 2 (NEWS2) upon admission, primary site of *E coli* bloodstream infection, type of antibiotic treatment for the bloodstream infection, admission to IMCU or ICU during in-patient care for the bloodstream infection, time to fever resolution, re-admissions and recurrence of *E coli* infection within 30 days of hospital admission, and mortality within 90 days of hospital admission and cause of death. At the time of clinical data collection, heteroresistance status of the isolates was unknown to the researchers. Because of the logistics of the data system implemented in the hospitals where the samples originated from, time between hospital admission and transfer to an IMCU or ICU could not be recorded. Clinical data for the entire study population are presented in appendix 1 (pp 4–7). In accordance with the approval by the ethical review board, data are only presented at the group level.

We focused on breakpoint crossing heteroresistance (BCHR), which means that the isolate is monoclonal, the main population does not grow at or above the clinical breakpoint as defined by The European Committee on Antimicrobial Susceptibility Testing (EUCAST) guidelines, the subpopulation grows at concentrations of antibiotics that are at least eight times higher than the main population and this concentration is at or above the clinical breakpoint, and the frequency of the resistant subpopulation is at least 1×10^–7^.^1–3^ Isolates were first screened on agar plates supplemented with cefotaxime, gentamicin, or piperacillin–tazobactam supplemented for the detection of subpopulations growing at the resistance breakpoint. If a subpopulation was detected, the BCHR phenotype was confirmed using a PAP test (appendix 1 pp 3–4). Raw data for PAP tests are in appendix 2. Minimal inhibitory concentrations (MICs) were determined by *E* tests as described in appendix 1 (p 3).

### Statistical analysis

Statistical analyses were performed using Matlab (Mathworks), R (version 4.4), and GraphPad Prism (version 9). Associations of BCHR phenotypes with worse clinical outcome were quantified in the form of relative risks (RRs) for worse clinical outcomes of patients suffering from a BCHR infection using χ^2^ tests. Correlation between BCHR phenotypes and the length of stay, length of empirical intravenous treatment, and time to fever resolution were investigated using Spearman’s correlation tests. To explore the associations between clinical outcomes and BCHR when patients are treated with the corresponding antibiotic, we used multivariate analyses (performed in GraphPad Prism) to estimate the odds ratios (ORs), hazard ratios (HRs), and 95% CIs while accounting for confounders. Specifically, for the effects of BCHR on treatment failure (switching or adding other drugs), 90-day mortality, recurrence of *E coli* infection within 30 days of admission to hospital, re-admission to hospital within 30 days of admission to hospital, and admittance to an IMCU or ICU, logistic regression models were fitted using sex, age, CCI, previous antibiotic treatment, NEWS2, and site of infection as explanatory variables. Interactions between the presence of BCHR for piperacillin–tazobactam with piperacillin–tazobactam treatment and presence of BCHR for gentamicin with gentamicin treatment were also included to investigate the effects of the BCHR phenotype when treated with the corresponding antibiotic.

The effects of BCHR on the length of stay in hospital, the time to fever resolution, and the type of antibiotic treatment were analysed as HRs to time-to-event outcomes using Cox regression models with the same explanatory variables. All multivariate logistic regression models had a Hosmer–Lemeshow p value above 0⋅4, indicating an acceptable fit. Cox regression models had concordance indices of 0⋅7 for the length of stay in hospital, 0⋅6 for the length of empirical intravenous treatment, and 0⋅6 for the time to fever resolution. An alternative statistical analysis using cohort segmentation is also presented in appendix 1 (pp 4–7, 14–16).

### Role of the funding source

The funders of the study had no role in study design, data collection, data analysis, data interpretation, orwriting of the report.

## Results

A total of 255 participants with corresponding *E coli* isolates met the inclusion criteria out of 500 assessed for eligibility. Using a pre-screen test and PAP tests appendix 2, we measured BCHR towards cefotaxime, gentamicin, and piperacillin–tazobactam (table 1, raw data are shown in appendix 2). The prevalence of BCHR varied extensively depending on the antibiotic. Only one (<1%) of 255 strains was BCHR for cefotaxime, whereas 109 (43%) of 255 strains were BCHR for gentamicin, and 22 (9%) of 255 strains were BCHR for piperacillin–tazobactam. The low prevalence of cefotaxime BCHR in this study did not allow for further statistical analysis on its underlying risk factors and clinical effect. We assessed how frequently the hospital laboratory in Uppsala, Sweden misclassified BCHR by disk diffusion. According to EUCAST guidelines, isolates with growth of colonies within the inhibition zone corresponding to clinical resistance should be classified as resistant. The clinical classification of BCHR strains revealed extensive misclassification (table 1). Specifically, the three antibiotics were misclassified 96% of the time with 120 (96%) of 125 isolates erroneously classified as susceptible and four (3%) of 125 isolates erroneously classified as intermediate. The very major error rate across the whole cohort was 47% due to BCHR (ie, 120 heteroresistant isolates misclassified as susceptible out of 255 isolates analysed).

Patient characteristics for the entire study population of 255 individuals are presented in appendix 1 (pp 7–14), including susceptibility towards the respective study drugs. No significant increases in RRs for a negative outcome were found without considering which treatment the patient received (table 2). Furthermore, the BCHR phenotypes had no correlation to the length of stay or time to fever resolution. This finding suggests that any treatment complications from BCHR relate to the strain’s heterogeneous treatment response, rather than other genetic or phenotypic differences. Using multivariate regression modelling, we then established whether BCHR is associated with adverse outcomes (table 3). Patients treated with gentamicin who had a gentamicin BCHR infection had 5⋅6 times higher odds for admittance to the ICU (95% CI 1⋅1–42⋅0, p=0⋅041) than patients in the corresponding control group (ie, patients treated with gentamicin who did not show gentamicin BCHR), indicating an RR of 5⋅3 (95% CI 1⋅2–45⋅0) for ICU admittance. Specifically, five (25%) of the 20 patients treated with gentamicin who showed gentamicin BCHR were admitted to the ICU, whereas none of the 18 patients treated with gentamicin who showed no gentamicin BCHR were admitted to the ICU. Additionally, the patients treated with gentamicin who showed gentamicin BCHR had 7⋅1 times higher odds of dying than those in the corresponding control group (95% CI 1⋅2–49⋅2, p=0⋅030), indicating an RR of 4⋅7 (95% CI 1⋅1–52⋅0) for mortality. Specifically, six (30%) of 20 patients treated with gentamicin who showed gentamicin BCHR died, whereas none of the 18 patients treated with gentamicin who showed no gentamicin BCHR died. No other increased risks were found to associate with gentamicin BCHR (table 3). All patients treated with gentamicin and admitted to the ICU received a β-lactam at admission, which the infected isolate was later reported as being susceptible to by disk diffusion assay.

The patients who showed piperacillin–tazobactam BCHR and were treated with piperacillin–tazobactamhad 3⋅1 times higher odds for admittance to the IMCU (95% CI 1⋅1–9⋅6, p=0⋅041) than patients in the corresponding control group (ie, the remainder of the cohort excluding those treated with gentamicin), indicating an RR of 2⋅4 (95% CI 1⋅1–8⋅2) for admittance to the IMCU. Specifically, two (25%) of eight patients with piperacillin–tazobactam BCHR who were treated with piperacillin–tazobactam were admitted to the IMCU, whereas nine (4%) of 204 patients from the corresponding control group were admitted to the IMCU. No additional increases in risk of any worse clinical outcome were detected (table 3). An alternative statistical analysis using cohort segmentation (appendix 1 pp 4–7, 14–16) gave similar conclusions to the multivariate analysis.

It is important to note that the multivariate regression analyses performed showed a statistically significant association between the BCHR phenotypes and clinical outcome only if one takes into account the treatment the patients received. Thus, if the analysis disregards which specific antibiotic the patient received, there was no association between BCHR phenotypes and worse clinical outcomes.

Table 4 summarises the comparison between patients who exhibited gentamicin BCHR and received gentamicin treatment and the control group. Table 5 summarises the comparison between patients who exhibited piperacillin–tazobactam BCHR and received piperacillin–tazobactam treatment and the control group. A notable difference is the proportion of patients with urinary tract infections (UTIs) in the group who showed gentamicin BCHR (seven [35%] of 20 patients) compared with the control group (17 [94%] of 18 patients; table 4). NEWS2 scores were also slightly elevated in patients who showed gentamicin or piperacillin–tazobactam BCHR compared with the respective control groups. Neither group who showed BCHR and received the corresponding antibiotic showed a significant difference in length of stay compared with the control group.

In an exploratory analysis, a strong association (p=0⋅0010, χ^2^ test for independence) was detected between previous β-lactam treatment within 90 days of admission to hospital and subsequent infection with a piperacillin–tazobactam BCHR strain, but no associations could be detected for gentamicin because previous aminoglycoside treatment was infrequent in this cohort. An additional risk factor for BCHR was pre-existing lymphoma, which was associated with the occurrence of piperacillin–tazobactam BCHR (p=0⋅0020, χ^2^ test for independence). However, there were not enough patients with lymphoma (n=2) in this cohort to investigate potential increases in risk of suffering from a BCHR infection. There was no other association between a piperacillin–tazobactam BCHR or gentamicin BCHR infection for patients with NEWS2 or CCI scores or the comorbidities used to calculate CCI.

We identified the MIC of piperacillin–tazobactam and gentamicin for all the strains to assess whether there was any correlation between MICs and BCHR status because it might be expected that strains with an MIC closer to the BCHR breakpoint might be more likely to produce a subpopulation above it. For piperacillin–tazobactam there was a weak correlation between MIC and BCHR status but not for gentamicin (appendix 1 p 21).

## Discussion

Here we report three major findings: (1) a high prevalence of heteroresistance in *E coli* bloodstream infections for gentamicin and piperacillin–tazobactam, but not for cefotaxime, (2) a high frequency of heteroresistance misclassification by standard antibiotic susceptibility testing, and (3) associations between BCHR to gentamicin and piperacillin– tazobactam and negative clinical outcomes. Previous reports on heteroresistance towards β-lactam antibiotics, specifically carbapenems, show similar frequencies and heteroresistance prevalences ranging from approximately 4% to 35% depending on the study.^16,18,19^ For gentamicin, previous work showed a heteroresistance prevalence of 67% among *E coli*, but most of these gentamicin-heteroresistant strains were strains with a resistant majority population.^4^ Overall, these data show that heteroresistance frequencies can be very high for some antibiotic and bacteria combinations, and underscores the need for a more systematic surveillance of the heteroresistant phenotype in patient isolates.

120 (96%) of the 125 BCHR strains were misclassified as susceptible using routine disk diffusion for antibiotic susceptibility testing with a very major error rate of 120 (47%) of 255 strains being misclassified as susceptible. This error rate is much higher than the very major error rate for standard susceptibility testing of piperacillin–tazobactam, which is typically below 2%.^20,21^ This finding suggests that standard antibiotic susceptibility tests are unsuitable to detect BCHR with meaningful consistency, and that the surveillance of BCHR is especially difficult.^22^ The reason for this unsuitability is the fundamental principle of disk diffusion assays and E-tests, which generate an inhibition zone that—depending on its size—classifies the tested strain as resistant or susceptible. If colonies are detected within the inhibition zone, EUCAST guidelines instruct that such isolates are classified as resistant for cefotaxime, gentamicin, and piperacillin–tazobactam.^23^ However, in standard antibiotic susceptibility testing practices, the inoculum sizes and the number of cells in the inhibition zone is often too small to detect the rare resistant subpopulations characteristic of BCHR.^3^

We report two key findings regarding clinical outcomes. First, if the analysis disregards which specific antibiotic the patient received, there was no association between BCHR phenotypes and worse clinical outcomes (table 2). This finding indicates that any treatment complications arising from BCHR are due to the strain’s heterogeneous treatment response, rather than other genetic or phenotypic differences that affect infection severity. Second, when comparing patients treated with antibiotics for which the *E coli* isolate is BCHR with patients receiving antibiotic to which the infecting isolate is non-heteroresistant, we found, a significantly increased risk of escalation of care to the ICU and IMCU, strongly associated with both BCHR towards gentamicin and piperacillin–tazobactam. Similarly, in the case of gentamicin, an increased risk of all-cause mortality associated with BCHR was also observed. However, we found no significant difference in the primary outcome of length of hospital stay. For secondary outcomes, there was no increased risk of switching to or adding another intravenous antibiotic among the patients with gentamicin or piperacillin–tazobactam BCHR infections who were treated with the respective antibiotic.

There are potential explanations for the differences observed between the different outcomes, and there are also limitations to this study. First, even though the negative secondary outcomes are statistically significant they are based on a small number of patients. Furthermore, our most significant results are based on gentamicin heteroresistance affecting the treatment outcomes of combination therapy, a treatment strategy whose efficiency has been questioned.^24^ In addition, there are always risks of unknown confounding factors when using retrospective data to compare patient groups. However, data on vital parameters and underlying comorbidities were collected at admission to account for the most probable confounders. We eliminated the risk of confirmation bias because the heteroresistant status of the isolates was unknown to the researcher collecting data from the medical records. However, there might exist other potential confounders. There are some indications that the patients who showed BCHR had more serious or different infections than the patients in the respective control groups. For gentamicin BCHR, seven (35%) of the 20 patients who showed gentamicin BCHR had a urinary source of infection compared with 17 (94%) of 18 patients in the corresponding control group. Additionally, NEWS2 scores were higher in patients who showed gentamicin BCHR than in patients in the corresponding control group, 7⋅8 (range 0–14⋅0) *vs* 5⋅9 (2⋅0–16⋅0). These differences indicate that the patients who showed gentamicin BCHR were more severely ill because both non-urinary sources of *E coli* bloodstream infections and elevated NEWS2 scores at admission are associated with increased mortality.^25,26^ Similarly, the patients who showed piperacillin–tazobactam BCHR and did not receive gentamicin had a higher mean NEWS2 score than those in the corresponding control group (6⋅0 *vs* 4⋅3), but the groups had a similar proportion of patients with a urinary tract focus of the bloodstream infection (seven [88%] of eight patients *vs* 148 [72%] of 204 patients). Piperacillin–tazobactam has a broader antimicrobial spectrum than cefotaxime and might have been given to patients who presented as more severely ill or who showed signs of deterioration. This difference could explain the increased risk of the patients who showed piperacillin–tazobactam BCHR being admitted to the IMCU compared with the corresponding control group. However, the BCHR phenotype was not associated with worse clinical outcome outside of the specific situation where treatment with the antibiotic in question was given.

The large proportion of UTIs is expected in a study of mainly community-acquired *E coli* bloodstream infections, which increases the generalisability of our results. However, UTIs are widely regarded as low inoculum infections with all three study antibiotics reaching high concentrations in urine. Thus, from a pharmacokinetics and pharmacodynamics perspective, these patients would probably be less vulnerable to suboptimal antibiotic treatment than those suffering from high inoculum infections with lower antibiotic concentrations in the target tissue (eg, pneumonia). It is plausible that heteroresistance could have a bigger effect on treatment outcomes in high-inoculum infections because of these pharmacokinetic and pharmacodynamic considerations. In addition, *E coli* bloodstream infections originating outside the urinary tract have a higher mortality rate. Thus, differing proportions of UTIs as the infection focus for bloodstream infections could affect the analysis when comparing the study groups.

Another weakness is the scarcity of data on how the timing of antibiotic treatment relates to different events. Although all the patients in our dataset were started on antibiotic treatment upon admission, some were switched to a drug other than the initial antibiotic or received an additional one during their hospital stay. Changing or adding antibiotics introduces uncertainty on the causal relationship between the treatments given and clinical outcomes.

Regarding the escalation of care to the ICU, our data do not include the timing of ICU admission and how it relates to gentamicin treatment. Because aminoglycosides are generally given to patients in an unstable condition or those with septic shock, patients might have been given gentamicin and moved to the ICU simultaneously. Also, because gentamicin is given in combination with other antibiotics, we cannot unambiguously claim that gentamicin is responsible for the worsening clinical outcome and not the partner drug, although all patients treated with gentamicin and admitted to the ICU received an initial β-lactam antibiotic to which the isolate would be reported to be susceptible using standard antibiotic susceptibility testing.

The method used to assess time until fever resolution (ie, timestamp on physician’s note and temperature checks at fixed hours without accounting for possible routine administration of antipyretic drugs) are imprecise and might have missed small but potentially significant differences. The imprecision of the method could explain why we saw no difference in this secondary outcome.

Furthermore, the study population consisted entirely of patients living in a specific health-care region in Sweden, which could affect the generalisability of our results. Thus, future studies should aim for a multicentre or international design. The sex and age distributions in our study were similar to larger previous studies on *E coli* bloodstream infections.^27^

Given the possible negative effect on clinical outcomes by BCHR and the risk of misclassifying the isolates as susceptible, it is also important to establish risk factors for infections with the BCHR phenotype. In line with earlier studies on Gram-negative bacteria, we found that previous exposure to antibiotics increased the risk for the present infection to be heteroresistant, similar to earlier studies of carbapenems and heteroresistance.^25,28^ The exact mechanisms behind the association of β-lactam treatment and subsequent infection with a piperacillin–tazobactam BCHR strain warrant future longitudinal studies to investigate the patient resistome and how it is shaped by treatment preceding the infection of interest.

Placing our results in a larger clinical context beyond our study drugs, one might look to novel treatment options for multidrug-resistant Enterobacteriaceae, for which heteroresistance seems to be quite common.^13,26^ There is an ongoing debate on the clinical significance of heteroresistance in these cases, which underscores the need for studies such as ours.^24^

In summary, this study of *E coli* BCHR in a clinical setting showed that the prevalence of BCHR varies considerably between different antibiotics and that regular antibiotic susceptibility testing methods are inadequate in identifying BCHR. Most importantly, BCHR to the treatment given was associated with increased mortality and admittance to ICU in the case of gentamicin and increased admittance to IMCU for piperacillin–tazobactam. Although these results must be considered in the context of the limitations discussed, our findings underscore worrisome clinical implications of BCHR phenotypes.

## Supplementary Material

1

2

## Figures and Tables

**Table 1:** Prevalence and clinical antimicrobial susceptibility testing classification of BCHR phenotypes for cefotaxime, gentamicin, and piperacillin-tazobactam

	BCHR prevalence	Susceptible	Intermediate	Resistant

Cefotaxime	1/255 (>1%)	0	1/1 (100%)	0
Gentamicin	109/255 (43%)	102/102 (100%)	0	0
Piperacillin-tazobactam	22/255 (9%)	18/22 (82%)	3/22 (14%)	(1/22) 5%

Data are n/N (%). The prevalence was based on 255 clinical isolates. Regarding antibiotic susceptibility of the BCHR strains, they were classified as susceptible, intermediate, or resistant by disk diffusion in the clinical microbiology laboratory. For gentamicin, results were available for 102 (94%) of 109 isolates. BCHR=breakpoint crossing heteroresistance.

**Table 2:** RR of the BCHR infections to cause worse clinical outcome irrespective of which treatment the patients received

	RR (95% CI)

Treatment failure (switch to another drug)	1·1 (0−2·8)
All-cause mortality within 90 days of admission	1·5 (0·1−1·8)
Recurrent *E coli* infection within 30 days of admission	1·4 (0·5−9·2)
Re-admission to hospital within 30 days of original admission	0·9 (0·4−3·3)
Admittance to ICU or IMCU	0·9 (0·3−3·0)
Length of stay	0·5 (0·2−1·2)
Length of empirical intravenous treatment	0·7 (0·4−2·4)
Time before fever resolution	0·9 (0·7−1·5)

BCHR=breakpoint crossing heteroresistance. ICU=intensive care unit.

IMCU=intermediary care unit. RR=relative risk.

**Table 3:** ORs and HRs of gentamicin and piperacillin-tazobactam BCHR infections to cause worse clinical outcome in patients treated with the respective antibiotics

	Gentamicin BCHR		Piperacillin-tazobactam BCHR	
	OR of worse outcome (95% CI)	p value	OR of worse outcome (95% CI)	p value

Treatment failure (switch to another drug)	1⋅0 (0⋅3−3⋅1)	p=0⋅98	0⋅9 (0⋅8−1⋅0)	p=0⋅96
All-cause mortality within 90 days of admission	7⋅1 (1⋅2−49⋅2)	p=0⋅030	1⋅0 (0⋅8−1⋅2)	p=0⋅41
Recurrent *E coli* infection within 30 days of admission	1⋅7 (0⋅7−3⋅8)	p=0⋅40	1⋅3 (0⋅5−1⋅5)	p=0⋅19
Re-admission to hospital within 30 days of original admission	1⋅0 (0⋅4−1⋅2)	p=0⋅58	0⋅7 (0⋅3−2⋅2)	p=0⋅59
Admittance to IMCU	1⋅1 (0−2⋅1)	p=0⋅92	3⋅1 (1⋅1−9⋅6)	p=0⋅041
Admittance to ICU	5⋅6 (1⋅1−42⋅0)	p=0⋅043	0⋅9 (0⋅5−2⋅1)	p=0⋅52
Length of empirical intravenous treatment*	1⋅16 (0⋅3−3⋅3)	p=0⋅34	1⋅1 (0⋅1−2⋅7)	p=0⋅72
Length of stay*	0⋅9 (0⋅3−2⋅1)	p=0⋅77	0 (0−1⋅1)	p=0⋅99
Time before fever resolution*	0⋅6 (0−1⋅7)	p=0⋅11	0⋅7 (0⋅6−1⋅6)	p=0⋅46

Outcomes with p<0⋅05 are effects for which the respective multivariate analysis yielded a statistically significant contribution to the outcome prediction of gentamicin BCHR when patients were treated with gentamicin, and piperacillin-tazobactam BCHR when patients were treated with piperacillin-tazobactam and not gentamicin. The terms gentamicin BCHR and piperacillin-tazobactam BCHR refer to the interaction terms of the multivariate model of the BCHR phenotypes with their corresponding treatments. All model outputs are in appendix 1 (pp16−21). BCHR=breakpoint crossing heteroresistance. HR=hazard ratio. OR=odds ratio. ICU=intensivecare unit. IMCU=intermediary care unit.

* Odds are presented as HRs.

**Table 4:** Baseline characteristics and outcomes of patients with gentamicin BCHR infections that were treated with gentamicin and those in the corresponding control group (ie, patients treated with gentamicin who did not show gentamicin BCHR)

	Gentamicin BCHR (n=20)	Control group (n=18)

Sex Female Male	7 (35%) 13 (65%)	9 (50%) 9 (50%)
CCI score	5⋅2 (1⋅1−10⋅0)	4⋅1 (0−10⋅0)
NEWS2	7⋅8 (0−14⋅0)	5⋅9 (2⋅0−16⋅0)
Urinary tract focus of bloodstream infection	7 (35%)	17 (94%)
Age, years	74 (30−85)	66 (25−95)
Admittance to ICU	5 (25%)	0
All-cause mortality within 90 days of admission	6 (30%)	0
Treatment failure (switch to other drug)	2 (10%)	5 (28%)
Recurrent *E coli* infection within 30 days of admission	0	1 (6%)
Re-admission to hospital within 30 days of original admission	1 (5%)	4 (22%)
Length of stay, h	335 (38−1224)	228 (75−1843)
Time before fever resolution, h	71 (18−211)	63 (5−259)

Data are n (%) or mean (range). BCHR=breakpoint crossing heteroresistance.

CCI=Charlson Comorbidity Index. ICU=intensive care unit. NEWS2=National Early Warning Score 2.

**Table 5:** Baseline characteristics and outcomes of patients with piperacillin-tazobactam BCHR infections that were treated with piperacillin-tazobactam and those in the corresponding control group (ie, the remainder of the cohort excluding those treated with gentamicin).

	Piperacillin-tazobactam BCHR (n=8)	Control group (n=204)

Sex		
Female	5 (63%)	113 (55%)
Male	3 (38%)	91 (45%)
CCI score	4⋅0 (3⋅0−8⋅0)	4⋅8 (0−13⋅0)
NEWS2	6⋅0 (2⋅0−11⋅0)	4⋅3 (0−16⋅0)
UTI focus of bloodstream infection	7 (88%)	148 (72%)
Age, years	75 (61−88)	72 (17−97)
Admittance to IMCU	2 (25%)	9 (4%)
All-cause mortality within 90 days ofadmission	0	18 (9%)
Treatment failure (switch to other drug)	0	36 (18%)
Recurrent *E coli* infection within 30 days of admission	0	10 (5%)
Re-admission to hospital within 30 days of original admission	0	8 (4%)
Length of stay, h	210 (62−504)	231 (9−1567)
Time before fever resolution, h	23 (1−60)	37 (1−290)

Data are n (%) or mean (range). BCHR=breakpoint crossing heteroresistance. CCI=Charlson Comorbidity Index. IMCU=intermediary care unit. NEWS2=National Early Warning Score 2.

## Data Availability

The individual participant data that underlie the results reported in this study, and the results of corresponding lab assays, are shared after de-identification (text, tables, figures, and the appendix).

## Research in context

### Evidence before this study

Antibiotic treatment failures can occur even when an infecting isolate is deemed susceptible to a given antibiotic. One proposed potential explanation for this is heteroresistance, which is characterised by a minor subpopulation of resistant bacterial cells that co-exists with a majority susceptible population. The clinical relevance of heteroresistance is not clear even though mathematical modelling and animal experiments suggest that it could play a significant role in treatment failures. PubMed searches during the period Jan 1, 1950, to Nov 22, 2024, with various permutations of the search terms (heteroresistance, *Escherichia coli*, bloodstream infection, prevalence, misclassification, clinical impact, treatment), showed that studies of prevalence, misclassification, and clinical consequences of heteroresistance of *E coli* bloodstream isolates had not been done previously.

### Added value of this study

The present study shows for the first time, to our knowledge, that heteroresistance is prevalent among *Escherichia coli* bloodstream isolates for the clinically important antibiotics gentamicin and piperacillin–tazobactam. In addition, heteroresistance is frequently misclassified by disk diffusion assays and, importantly, associated with treatment complications (gentamicin and piperacillin–tazobactam) and increased mortality (gentamicin).

### Implications of all the available evidence

The frequent misdiagnoses of heteroresistance combined with the negative clinical outcomes highlight the need to better diagnose and manage infections caused by these common bacteria. We suggest that increased testing of heteroresistance for *E coli* bloodstream infections is needed.
