# Supplementary material for: Prevalence, misclassification, and clinical consequences of the heteroresistant phenotype in Escherichia coli bloodstream infections in patients in Uppsala, Sweden: a retrospective cohort study
Source: Lancet Microbe. Author manuscript; Available in PMC 2025 Apr 17. (PMC12004506; doi:10.1016/j.lanmic.2024.101010)

THE LANCET
Microbe

Supplementary appendix 1

This appendix formed part of the original submission and has been peer reviewed.
We post it as supplied by the authors.

Supplement to: Heyman G, Jonsson S, Fatsis-Kavalopoulos N, et al. Prevalence, misclassification, and clinical consequences of the heteroresistant phenotype in *Escherichia coli* bloodstream infections in patients in Uppsala, Sweden: a retrospective cohort study. *Lancet Microbe* 2025. https://doi.org/10.1016/j.lanmic.2024.101010

Appendix 1

Table of content

	Page
MIC test of isolates for TZP and GEN susceptibility.	2
Media and Antibiotics.	2
Break Point Crossing Heteroresistance prevalence and criteria.	2
Plate screening.	2-3
E-test.	3
Population Analysis Profile (PAP) test.	3-4
Alternative statistical analysis.	4-7
Table S1. Patient characteristics, antibiotic treatment, bacterial susceptibility and treatment outcomes at group level for study population of 255 patients.	7-9
Table S2: MIC test results performed post hoc in all isolates included in the study with Etests for TZP and GEN.	9-14
Table S3: Relative risks of GEN- and TZP- BCHR infections to cause worse clinical outcome in patients treated with the respective antibiotics.	14
Table S4: Comparison between GEN BCHR patients treated with GEN and their control group.	15
Table S5: Comparison between TZP BCHR patients treated with TZP and their control group (all the remaining patients not treated with GEN).	15-16
Table S6: Output of the multivariate models fit.	16-21
Figure S1. Illustration of GEN BCHR status of strains in relation to their MIC concentrations.	21
Figure S2. Illustration of TZP BCHR status of strains in relation to their MIC concentrations.	21

MIC test of isolates for TZP and GEN susceptibility

Isolates were streaked on MHA plates and grown overnight at 37°C. For each isolate, one colony was picked and grown for 26h in 1mL MHB in 10mL plastic vials at 37°C under vigorous agitation. Cultures were diluted 1:20 in 1xPBS. A sterile cotton swab was dipped in the suspension and used to spread the bacteria on MHA plates. Etests strips were put on the plates in opposite orientations, and the plates were incubated for 18h at 37°C before reading the results and taking pictures of the plates. Etests LOT numbers used: TZP LOT 10106112580, GEN LOT 1010523680 up to DA63988, LOT 1009194990 from DA64000. Results are tabulated in Table S2 and illustrated in supplementary Figures S1 and S2.

Media and Antibiotics

Mueller-Hinton (Difco) medium was used for growth in broth and for agar plates. Antibiotics (piperacillin, tazobactam, cefotaxime and gentamicin) were purchased from Sigma-Aldrich. For the piperacillin-tazobactam combination, a ratio of 8:1 (piperacillin:tazobactam) was used and stated concentrations refers to piperacillin concentration.

Break Point Crossing Heteroresistance prevalence and criteria

For a strain to be classified as BCHR to one of the studied antibiotics it needed to meet the following criteria in at least two population analysis profile (PAP) test replicates: (i) the bacterial isolate should be monoclonal, arising from a single cell, and grow in Mueller-Hinton media, (ii) the highest antibiotic concentration that has no effect on its growth should lie below the clinical breakpoint for resistance for the antibiotic in question. A reduction in growth >80% is considered an effect, (iii) the frequency of the resistant subpopulation should be $\geq 10^{-7}$ at or above the breakpoint concentration and (iv) the resistant population should grow on a ≥8-fold higher antibiotic concentration compared to the main population.

Plate screening

To detect possible subpopulations growing at antibiotic concentrations close to the clinical resistance break point (https://www.eucast.org/clinical_breakpoints) valid 2014-2015: piperacillin-tazobactam resistant (R) >16 mg/L, cefotaxime R >2 mg/L, gentamicin R >4 mg/L), all 255 isolates were plated on 16 mg/L piperacillin-tazobactam, 2 mg/L cefotaxime and 4 mg/L gentamicin. Bacteria from frozen 10% DMSO stocks were streaked on Mueller Hinton

(MH) agar plates and incubated at 37°C overnight. Single colonies were inoculated in 100 µL MH broth, incubated at 37°C, 199 rpm agitation, overnight. For a second overnight cultures the first overnight culture was diluted in phosphate buffered saline (PBS) 1:1000. 1 µL of the diluted culture were then inoculated in 100 µL or 1 mL MH broth and incubated at 37°C, 199 rpm agitation, overnight. These cultures were then diluted 1:20 in PBS, and 100 µL was spread on MH agar plates supplemented with antibiotics (16 mg/L TZP, 2 mg/L CTX and 4 mg/L GEN), using glass beads. Agar plates were incubated at 37°C for 48 h and colonies were counted. The pre-screen was performed in duplicate for all isolates.

E-test

E-tests (Biomerieux) were performed for isolates growing as lawns at 2 mg/L CTX when resistance of the main population was suspected. For the CTX E-test 50 µl overnight culture was diluted in 950 µl PBS and spread on a MH plate with a cotton swab. For GEN all strains were E-tested. 40 µl overnight culture was diluted in 280 µl PBS and spread on a MH plate with a cotton swab. Plates were incubated for 18h ±2 h at 37°C.

Population Analysis Profile (PAP) test.

Isolates with growth of ≥ 1 colony on the TZP, CTX and GEN containing screen plates (see Plate screen), as well as an MIC value (E-test) below the resistance breakpoint crossing (EUCAST guidelines year 2014/2015) (https://www.eucast.org/clinical_breakpoints) for CTX and GEN, were PAP tested for the respective AB. For the PAP test, overnight cultures started from one single colony were used. Cultures were serially diluted in PBS and plated on MH agar plates with and without AB supplementation. For all antibiotics 2-fold increments of antibiotics were used within the following antibiotic range: TZP from 0.25 to 128 mg/L, CTX from 0.016 to 4 mg/L and for GEN from 0.125 to 8 mg/L.

For low concentration of ABs (TZP 0.25 - 4 mg/L, CTX 0.016 - 0.5 mg/L, GEN 0.125 - 1 mg/L) and agar plates without ABs, triplicates of 5 µL drops of suitable dilutions were placed on agar plates to allow for a wider range of countable colonies on less plates. For higher AB concentrations of TZP (8-128 mg/L), CTX (1 to 4 mg/L) and GEN (2 to 8 mg/L), 100 µL of an 1:20 dilution, or subsequent 1:10 dilutions, of the overnight culture in PBS were spread on a full agar plate using glass beads (TZP and CTX) or with a sterile loop on half of an agar plate (GEN), in order to reduce the inoculum effect (i.e. increased colony count resulting from high

cell density and not individual cell resistance. Plates supplemented with ABs were incubated at 37°C overnight (drops) or 48 h (full plates), and plates without ABs were incubated at 30°C overnight. The shorter incubation and lower temperatures were used to prevent overcrowding on the plate and fusion of colonies. Frequencies of resistance were calculated by the ratio of number of colonies on plates with and without antibiotics. PAP test was performed in triplicates for TZP and CTX, and in duplicates for GEN. If a discrepancy in HR classification was observed for GEN, a third replicate was performed.

Alternative statistical analysis.

As an alternative to the multivariate analysis, we also performed a cohort segmentation to account for confounders. In this analysis, patients were sub-divided into cohorts that had received the same treatment and then analyzed with regard to risk for worse outcome (odds-ratio analysis).

Non-binary parameters were stratified as described below.

Stratification of length of stay: The median value of 139h was chosen as the threshold between what was subsequently considered a 'longer than average' length of stay.

Stratification of time to fever resolution: The median value of 30h was chosen as the threshold between what was subsequently considered a 'longer than average' time to fever resolution.

Stratification of age: A median value of 71 years was chosen as the threshold between what was subsequently considered a 'older than average' age.

Stratification of duration of treatment: The population exhibited 2 peaks one at 70h and one at 150 h, the in-between value of 130h was chosen as the threshold between what was subsequently considered a 'longer than average' duration of treatment.

Odds ratios analysis: Odds ratios for worse outcomes on patients infected with an BCHR isolate were calculated for the following parameters when applicable: Switching or adding another antibiotic (if not part of standard clinical practice), mortality, relapse, readmission, admission to the ICU or IMCU, length of stay, time to fever resolution and duration of treatment. Matlab was used to segregate the data into the 4 categories for both types of BCHR (GEN or TZP) and all clinical outcomes separately.

For analysis of TZP BCHR the categories were:

- *Category 1: patients infected with TZP BCHR strains and treated with TZP with worse clinical outcomes.*

- *Category 2: patients infected with TZP BCHR strains and treated with TZP with no worse clinical outcome.*
- *Category 3: Patients not infected with TZP BCHR strains and treated with TZP, with worse clinical outcome.*
- *Category 4: Patients not infected with TZP BCHR strains and treated with TZP, with no worse clinical outcome.*

For analysis of GEN BCHR the categories were:

- *Category 1: patients treated with GEN and infected with GEN BCHR strains with worse clinical outcomes.*
- *Category 2: patients treated with GEN and infected with GEN BCHR strains with no worse clinical outcome.*
- *Category 3: patients treated with GEN and not infected with GEN BCHR strains with worse clinical outcome.*
- *Category 4: patients treated with GEN and not infected with GEN BCHR strains with no worse clinical outcome*

Relative risks for worse clinical outcome were calculated independently for all outcomes and BCHR types, as (CAT1/CAT2) /(CAT3/CAT4). In the cases where a category had a count of 0 approximate relative risk was calculated by adding 1 to the counts of all categories, Fisher's exact tetsts and subsequent p values were calculated on the original dataset, the approximate relative risk was used only for reporting. To protect against statistical bias and reduce the probability of false positives only relative risks that were higher than 1.5 or lower than 0.5 were post hoc tested with a statistical hypothesis test (chi square or Fisher's exact) to establish significance using graphpad prism.

Risk factor analysis: Chi square tests between established risk factors and the in vitro detected BCHR phenotype were conducted in matlab and graphpad prism

Confounders: Relative risks analyses and correlation analyses were conducted as described above in matlab and graphpad prism

We proceeded to analyze each BCHR phenotype separately for each antibiotic. Since patient selection for the cohort was agnostic to the HR status of the isolates, the effects of BCHR to clinical metrics were investigated by calculating the relative risk for a worse clinical outcome a BCHR patient would have compared to a non BCHR patient in the same sub-cohort. Patient

characteristics for the entire study population of 255 individuals are presented in supplementary Table S1, including susceptibilty towards the respective study drugs as reported in the medical health records.

(i) Accounting for potential confounding factors

No correlation between BCHR phenotypes and risks of worse clinical outcomes was found without taking into account what treatment the patient received. This suggests that any treatment complications from BCHR relate to the strain's heterogeneous treatment response, rather than other genetic or phenotypic differences (for example in virulence).

GEN treatment emerged as a significant confounder. Patients receiving GEN, often prescribed for severe BSIs had a 7-fold higher risk of ICU admission ($p<0.001$, for GEN-treated patients: 5 out of 43 admitted to the ICU; for non-GEN-treated patients: 3 out of 212 admitted to ICU). Therefore, a sub-cohort of GEN-treated patients was analyzed separately, also excluding those with GEN-resistant strains. This sub-cohort exclusion was crucial to ensure that the influence of BCHR on outcomes was not confounded by either more severe BSIs or undetected GEN resistance. GEN-treated patients were also excluded from the analysis of TZP BCHR outcomes.

The division into two sub-cohorts based on GEN treatment introduced another confounding factor related to ICU and IMCU admissions. Many patients needing ICU care received GEN, leading to their exclusion from the TZP BCHR analysis. This created a confounding effect on the impact of TZP BCHR on ICU admissions. Similarly, the influence of GEN BCHR on IMCU admissions was confounded, as GEN-treated patients were often more severely ill and transferred to the ICU. To address these confounders, the study excluded the analysis of GEN BCHR's impact on IMCU admission and TZP BCHR's impact on ICU admission.

Lastly, the study found a weak correlation between older age and TZP BCHR ($r=0.14$ $p=0.02$), but no significant correlation with GEN BCHR. Despite older age being weakly associated with getting a TZP BCHR infection, there was no discernible increase in relative risk of getting such an infection. Therefore, age alone cannot explain the observed increases in risk for worse outcomes associated with TZP and GEN BCHR.

(ii) Correlations of BCHR with increased risks of adverse clinical outcomes

Using the sub-cohorts described above, we then proceeded to determine if BCHR is associated with adverse outcome. For patients treated with GEN with a BCHR infection, there was 4.4-fold significant increase in relative risk of admittance to the ICU (95% CI 1 to 33, $p=0.02$) with

5 out of 20 GEN BCHR patients being admitted to intensive care compared to 0 out of 18 patients that showed no BCHR to GEN. Additionally, the GEN BCHR infected patients treated with GEN had a 5-fold significant increase in relative risk of mortality. (95% CI 1 to 30, p=0.03), with 6 out of 20 GEN BCHR patients dying compared to 0 out of 18 that showed no BCHR to GEN. No other increased risks were found to correlate with GEN BCHR (Table S3).

The TZP BCHR infected patients that received TZP but not GEN had a 4.7-fold significant (95% CI 1 to 15, p=0.02) increase in relative risk for admittance to an IMCU with 2 out of 8 TZP BCHR patients being admitted to the IMCU compared to 9 out of 204 patients in the entire cohort that showed no BCHR to TZP. No additional increases in risk of any worse clinical outcome were detected (Table S3).

Tables S4 and S5 summarizes the comparison between the BCHR patients treated with the antibiotic in question and their control groups. A notable difference is the proportion of UTIs in the GEN BCHR groups which was 35% compared to 94.4% in the control group as well as slightly elevated NEWS2 scores in the GEN BCHR and TZP BCHR groups. None of the BCHR groups treated with the corresponding antibiotic showed a significant difference in length of stay compared to the control group. Table S6 shows the output of the multivariate models fit

Table S1. Patient characteristics, antibiotic treatment, bacterial susceptibility and treatment outcomes at group level for study population of 255 patients.

**Patient characteristics**	
Sex, female, n (%)	135 (52.9)
Age, median (IQR), years	75 (65 - 85)
CCI, median (IQR)	5.2 (3 - 6)
Myocardial infarction history, n (%)	53 (20.8)
Congestive heart failure, n (%)	48 (18.9)
Vascular disease, n (%)	14 (5.5)
Cerebrovascular disease, n (%)	49 (19.2)
Dementia, n (%)	32 (12.5)
COPD, n (%)	17 (6.7)
Connective tissue disease, n (%)	6 (2.4)

Peptic ulcer, n (%)	12 (4.7)
Liver disease, n (%)	9 (3.5)
Diabetes mellitus, n (%)	71 (27.8)
Hemiplegia, n (%)	7 (2.7)
Kidney failure, n (%)	8 (3.1)
Solid tumour, n (%)	43 (16.9)
Leukemia, n (%)	3 (1.2)
Lymphoma, n (%)	1 (0.4)
**At BSI admission**	
Previous Antibiotics within 90 days, n (%)	110 (43.1)
Beta lactam antibiotics within 90 days, n (%)	81 (31.8)
NEWS2 on admission, median (IQR)	5 (2 - 7)
Urinary focus of BSI, n (%)	198 (77.6)
**Susceptibility of BSI isolates**	
CTX S, n (%)	238 (93.3)
TZP S, n (%)	238 (93.3)
GEN S, n (%)	218 (91.6)
**Treatment for BSI**	
CTX treated, n (%)	124 (48.6)
TZP treated, n (%)	133 (52.2)
GEN treated, n (%)	59 (23.21)
Other antibiotics, n (%)	41 (16.1)
**Clinical outcomes**	
Hospital stay, median (IQR), days	5.9 (3.8 - 10.8)
Time to fever resolution, median (IQR), hours	26.3 (5.6 - 54.2)
Admission to ICU, n (%)	8 (3.1)
Admission to IMCU, n (%)	35 (13.7)

Switching IV antibiotic during BSI treatment, n (%)	24 (9.4)
Adding another antibiotic to BSI treatment, n (%)	26 (10.2)
Mortality within 90 days, n (%)	25 (9.8)
Readmission within 30 days, n (%)	34 (13.3)
Relapse of *E. coli* infection within 30 days, n (%)	11 (4.3)

Table S2: MIC test results performed post hoc in all isolates included in the study with Etests for TZP and GEN. Main indicates MIC value for main population.

	**MIC TZP main**	**MIC GEN main**
DA62890	1.5	0.75
DA62892	0.75	0.5
DA62896	1	0.5
DA62900	1	0.5
DA62902	1	0.38
DA62904	1	0.38
DA62906	1	1
DA62908	1.5	0.38
DA62910	1.5	0.25
DA62918	2	0.75
DA62920	2	0.75
DA62924	1	0.75
DA62926	1	0.38
DA62928	3	0.75
DA62930	0.75	2
DA62932	1	0.5
DA62934	1.5	0.75
DA62938	1	0.38
DA62940	1.5	1
DA62944	2	0.25
DA62948	1.5	0.38
DA62958	2	0.5
DA62960	1	0.38
DA62962	1.5	0.75
DA62974	2	0.38
DA62976	1.5	1.5
DA62978	16	16
DA62986	2	0.38
DA62996	1	0.5
DA62998	3	1
DA63000	1	0.5
DA63002	1.5	1
DA63004	1	0.19
DA63010	1	0.38

DA63014	1	0.5
DA63020	2	0.38
DA63032	0.75	0.25
DA63034	2	0.38
DA63036	1.5	0.5
DA63038	1.5	0.38
DA63048	1	0.38
DA63050	0.75	0.38
DA63056	2	0.5
DA63058	1	0.38
DA63060	1	0.75
DA63062	2	0.5
DA63068	0.38	0.75
DA63072	1.5	1
DA63074	1.5	1
DA63078	0.5	0.25
DA63080	2	0.38
DA63086	12	0.38
DA63090	0.75	0.75
DA63094	0.5	1
DA63112	1.5	0.5
DA63118	0.75	0.5
DA63120	0.75	0.38
DA63128	0.75	0.75
DA63132	2	0.38
DA63134	0.75	0.38
DA63136	1	0.25
DA63140	0.75	0.38
DA63144	1.5	0.25
DA63146	1.5	0.75
DA63148	1	0.38
DA63150	16	48
DA63154	2	0.5
DA63158	2	32
DA63164	2	128
DA63166	1	0.38
DA63168	1	0.38
DA63170	1	0.5
DA63172	2	0.38
DA63174	0.75	0.5
DA63176	1.5	0.75
DA63178	2	0.5
DA63180	3	0.5
DA63182	1.5	0.38
DA63184	2	0.25
DA63186	1.5	0.5
DA63188	1	0.5
DA63190	1.5	0.75
DA63192	2	0.38
DA63196	1.5	0.38
DA63200	0.75	1

DA63208	1	0.75
DA63218	1.5	0.75
DA63236	3	1.5
DA63240	1.5	0.38
DA63246	1	0.38
DA63254	1.5	0.5
DA63258	1	0.38
DA63260	1.5	0.5
DA63264	1	0.25
DA63266	12	0.5
DA63270	1.5	0.5
DA63276	1.5	0.5
DA63278	1.5	0.25
DA63282	1	0.75
DA63290	1	2
DA63294	1.5	0.75
DA63296	0.75	0.5
DA63302	0.75	0.38
DA63306	1	48
DA63308	3	0.5
DA63310	1.5	1
DA63312	1	0.25
DA63318	1.5	0.5
DA63320	24	24
DA63322	6	0.5
DA63324	1.5	0.75
DA63326	0.75	0.5
DA63332	4	0.75
DA63334	1	0.75
DA63338	2	0.5
DA63342	24	48
DA63362	2	0.5
DA63364	0.75	0.38
DA63368	1	0.5
DA63370	1.5	0.75
DA63376	0.75	0.75
DA63378	1	1
DA63382	1	0.75
DA63384	0.75	0.75
DA63568	1	0.5
DA63570	1	0.75
DA63572	1.5	0.5
DA63582	1.5	0.75
DA63584	0.75	0.38
DA63588	2	0.38
DA63590	1	0.75
DA63594	2	0.38
DA63596	1.5	0.5
DA63598	1.5	0.5
DA63600	2	0.38
DA63606	0.5	0.38

DA63608	12	1
DA63610	1	0.75
DA63616	1.5	0.75
DA63618	3	0.75
DA63622	1	0.25
DA63624	1	0.5
DA63630	1.5	0.75
DA63636	0.75	0.38
DA63640	1.5	0.38
DA63642	1.5	0.38
DA63644	1	0.5
DA63650	0.75	0.38
DA63654	1.5	64
DA63656	1.5	0.75
DA63660	4	0.5
DA63662	1	0.75
DA63664	0.75	0.25
DA63668	0.5	0.5
DA63672	1	0.75
DA63676	24	0.38
DA63680	1.5	1
DA63682	1.5	0.38
DA63686	0.75	0.38
DA63690	2	64
DA63694	2	0.25
DA63696	1.5	0.5
DA63698	1	0.38
DA63708	0.5	0.25
DA63710	1.5	0.25
DA63716	1	0.25
DA63722	1	0.5
DA63724	2	0.5
DA63726	2	24
DA63734	1	0.19
DA63742	1.5	1
DA63746	1	0.25
DA63748	0.5	1
DA63758	0.75	0.38
DA63760	48	48
DA63762	1.5	0.25
DA63766	16	0.38
DA63768	1.5	0.19
DA63772	8	48
DA63776	3	0.75
DA63782	1.5	1
DA63784	16	1
DA63786	0.75	0.25
DA63788	0.75	48
DA63796	1	0.5
DA63798	0.5	0.38
DA63802	0.75	0.75

DA63804	1.5	1
DA63820	1.5	1
DA63822	1.5	32
DA63826	2	0.38
DA63828	3	0.38
DA63830	1.5	0.5
DA63832	2	0.38
DA63842	2	0.5
DA63844	1	0.38
DA63846	0.75	0.5
DA63848	1	0.5
DA63852	0.75	0.5
DA63854	0.5	0.38
DA63856	1	1
DA63858	1.5	0.75
DA63860	1	0.5
DA63862	3	32
DA63864	1.5	0.75
DA63868	3	1
DA63870	2	0.5
DA63874	1.5	0.75
DA63876	0.75	0.38
DA63878	1	0.25
DA63880	2	0.5
DA63882	1	0.38
DA63884	6	1
DA63886	1.5	0.38
DA63888	1.5	32
DA63892	1.5	0.5
DA63894	1	0.75
DA63900	1	0.5
DA63902	1.5	0.75
DA63904	1.5	0.5
DA63912	1.5	0.19
DA63916	1	0.5
DA63918	1.5	0.5
DA63922	2	0.75
DA63924	0.75	0.5
DA63936	0.38	0.5
DA63946	1	0.75
DA63948	2	1
DA63950	2	0.5
DA63954	1	0.38
DA63956	0.75	0.75
DA63970	1	0.75
DA63976	0.75	0.75
DA63978	0.5	1
DA63982	0.38	0.5
DA63986	0.5	0.19
DA63988	16	0.38
DA64000	1.5	0.5

DA64002	6	1
DA64008	1	0.38
DA64010	1.5	0.38
DA64022	0.5	0.38
DA64024	1.5	0.38
DA64026	3	16
DA64028	1.5	0.5
DA64030	1	24
DA64032	1.5	0.38
DA64036	1	0.25
DA64038	0.094	0.25
DA64040	2	0.5
DA64042	3	0.38
DA64044	1	0.75
DA64050	2	0.25
DA64058	2	0.75
DA64060	2	0.75

Table S3: Relative risks of GEN- and TZP- BCHR infections to cause worse clinical outcome in patients treated with the respective antibiotics. In bold are statistically significant results (Fisher's exact test) and + signs indicate approximate relative risks where absolute relative risks could not be calculated. NA indicates non applicable parameters (as explained in the confounders section)

**Clinical outcomes**	**Relative risk of worse outcome**	
	GEN BCHR	TZP BCHR
Treatment failure	0.6	0.6^{+}
All-cause mortality within 90 days	**5.0^{+} (p=0.03)**	1.1^{+}
Recurrent E coli infection within 30 days	1.4^{+}	1.8^{+} (p=0.5)
Re-admission to hospital within 30 days of original admission	0.4 (p=0.33)	0.7^{+}
Admittance to IMCU	NA	**4.7 (p=0.02)**
Admittance to ICU	**4.4^{+} (p=0.02**)	NA
Longer length of stay	1.0	1.2
Longer time before fever resolution	NA	1.1

Table S4: Comparison between GEN BCHR patients treated with GEN and their control group.

**GEN treated patients (38)**	**GEN BCHR**<br>**20 (100%)**	**Non GEN BCHR**<br>**18 (100%)**
Male	13(65%)	9.0 (50%)
CCI	5.2	4.1
NEWS2	7.8	5.9
UTI focus	7(35%)	17(94%)
Average age	74	66
ICU admittance	5 (25%)	0 (0%)
All-cause mortality within 90 days	6 (30%)	0 (0%)
Treatment failure (switch to other drug)	2 (10%)	5 (28%)
Recurrent *E coli* infection within 30 days	0 (0%)	1 (5.6%)
Re-admission to hospital within 30 days of original admission	1 (5.0%)	4 (22%)
Average length of stay (hours)	335	228
Average time before fever resolution (hours)	71	63

Table S5: Comparison between TZP BCHR patients treated with TZP and their control group (all the remaining patients not treated with GEN).

**Non GEN treated patients (212)**	**TZP BCHR**<br>**8 (100%)**	**Control group**<br>**204 (100%)**
Male	3 (38%)	91 (45%)
CCI	4	4.8
NEWS2	6	4.3
UTI focus	7 (88%)	148 (72%)
Average age	75	72
IMCU admittance	2 (25%)	9 (4%)
All-cause mortality within 90 days	0	18 (9%)
Treatment failure (switch to another drug)	0	36 (18%)
Recurrent *E coli* infection within 30 days	0	10 (4.9%)
Re-admission to hospital within 30 days of original admission	0	8 (3.9%)
Average length of stay (hours)	210	231

Average time before fever resolution (hours)	23	37

Table S6: Output of the multivariate models fit. MLR refers to multivariate logistic regression with corresponding Odds ratios and CR refers to cox regressions with corresponding hazards ratios. CI is confidence interval.

**Model output parameter**	**Model type**	**Variable (input parameter)**	**Parameter estimate**	**Sig. diff. than zero?**	**Odds/Hazard ratio (estimated)**	**95% CI of Odds/Hazard ratio**
IMCU admission	MLR	Intercept	-1.411	0.1712	0.244	0.02950 to 1.745
IMCU admission	MLR	pretreatment[1]	-0.5999	0.194	0.5489	0.2129 to 1.325
IMCU admission	MLR	sex[F]	0.4117	0.3478	1.509	0.6469 to 3.663
IMCU admission	MLR	cci	0.1717	0.0983	1.187	0.9662 to 1.457
IMCU admission	MLR	age	-0.04231	0.015	0.9586	0.9258 to 0.9917
IMCU admission	MLR	news2	0.2561	<0.0001	1.292	1.144 to 1.473
IMCU admission	MLR	GEN HR[1] : Treatment[2]	0.1292	0.9219	1.138	0.04569 to 12.08
IMCU admission	MLR	TZP HR[1] : Treatment[1]	1.133	0.0418	3.105	1.0673 to 9.567
ICU admission	MLR	Intercept	1.281	0.4472	3.6	0.1526 to 129.1
ICU admission	MLR	pretreatment[1]	1.609	0.1525	4.996	0.7786 to 99.36

ICU admission	MLR	sex[M]	-0.7296	0.3567	0.4821	0.08898 to 2.209
ICU admission	MLR	cci	-0.007114	0.9736	0.9929	0.6672 to 1.585
ICU admission	MLR	age	0.02459	0.4313	1.025	0.9621 to 1.090
ICU admission	MLR	news2	-0.07355	0.5234	0.9291	0.7385 to 1.167
ICU admission	MLR	GEN HR[1] : Treatment[2]	1.731	0.0437	5.647	1.123 to 42.18
ICU admission	MLR	TZP HR[1] : Treatment[1]	-0.00132	0.523	0.9301	0.653 to 1.322
Mortatlity	MLR	Intercept	-14.09	<0.0001	7.589E-07	2.874e-010 to 0.0003392
Mortatlity	MLR	pretreatment[1]	1.609	0.0149	4.997	1.480 to 20.90
Mortatlity	MLR	sex[F]	0.07253	0.9015	1.075	0.3388 to 3.485
Mortatlity	MLR	cci	0.585	<0.0001	1.795	1.384 to 2.449
Mortatlity	MLR	age	0.08004	0.0345	1.083	1.010 to 1.174
Mortatlity	MLR	news2	0.04275	0.5808	1.044	0.8934 to 1.216
Mortatlity	MLR	GEN HR[1] : Treatment[2]	1.968	0.0359	7.16	1.159 to 49.15
Mortatlity	MLR	TZP HR[1] : Treatment[1]	0.05386	0.4103	1.043	0.978 to 1.112

treatment failure	MLR	Intercept	11.48	0.0349	11.48	1.331 to 130.5
treatment failure	MLR	pretreatment[1]	0.8751	0.7722	0.8751	0.3544 to 2.203
treatment failure	MLR	sex[F]	0.6003	0.2774	0.6003	0.2284 to 1.477
treatment failure	MLR	cci	0.9296	0.525	0.9296	0.7467 to 1.176
treatment failure	MLR	age	1.013	0.4775	1.013	0.9761 to 1.051
treatment failure	MLR	news2	0.9298	0.2698	0.9298	0.8176 to 1.061
treatment failure	MLR	GEN HR[1] : Treatment[2]	0.9677	0.9784	0.9677	0.2250 to 6.902
treatment failure	MLR	TZP HR[1] : Treatment[1]	0.9155	0.955	0.9155	0.134 to 4.301
Relapse	MLR	Intercept	14.16	<0.0001	1414296	2820 to 4072311419
Relapse	MLR	pretreatment[1]	-1.325	0.0269	0.2658	0.07592 to 0.8233
Relapse	MLR	sex[F]	-0.09492	0.8694	0.9094	0.2847 to 2.826
Relapse	MLR	cci	-0.5699	<0.0001	0.5656	0.4167 to 0.7305
Relapse	MLR	age	-0.08729	0.0215	0.9164	0.8454 to 0.9828
Relapse	MLR	news2	-0.09486	0.1902	0.9095	0.7874 to 1.050

Relapse	MLR	GEN HR[1] : Treatment[2]	0.5041	0.4073	1.655	0.4846 to 5.424
Relapse	MLR	TZP HR[1] : Treatment[1]	0.0183	0.1914	1.323	0.5210 to 4.364
Readmittance	MLR	Intercept	2.288	0.0312	9.858	1.340 to 88.93
Readmittance	MLR	pretreatment[1]	-0.528	0.1871	0.5898	0.2660 to 1.292
Readmittance	MLR	sex[F]	-0.3606	0.3651	0.6973	0.3121 to 1.506
Readmittance	MLR	cci	-0.0128	0.903	0.9873	0.8089 to 1.225
Readmittance	MLR	age	-0.0004637	0.9781	0.9995	0.9662 to 1.033
Readmittance	MLR	news2	0.03379	0.5812	1.034	0.9205 to 1.172
Readmittance	MLR	GEN HR[1] : Treatment[2]	-0.3216	0.5985	1.079	0.2370 to 2.744
Readmittance	MLR	TZP HR[1] : Treatment[1]	0.07577	0.5883	0.723	0.4323 to 2.523
Treatment Length	CR	pretreatment[1]	1.006	0.9664	1.006	0.7599 to 1.327
Treatment Length	CR	sex[F]	0.8859	0.369	0.8859	0.6804 to 1.155
Treatment Length	CR	cci	0.9472	0.1435	0.9472	0.8796 to 1.017
Treatment Length	CR	age	0.9993	0.904	0.9993	0.9880 to 1.011

Treatment Length	CR	news2	0.9925	0.6974	0.9925	0.9548 to 1.031
Treatment Length	CR	GEN HR[1] : Treatment[2]	1.162	0.3472	1.162	0.8534 to 1.599
Treatment Length	CR	TZP HR[1] : Treatment[1]	1.086	0.7249	1.086	0.6670 to 1.677
Length of Stay	CR	pretreatment[1]	0.6248	0.1869	1.868	0.7319 to 4.806
Length of Stay	CR	sex[F]	0.3356	0.5117	1.399	0.5275 to 4.002
Length of Stay	CR	cci	-0.006115	0.9526	0.9939	0.8001 to 1.202
Length of Stay	CR	age	-0.01992	0.2318	0.9803	0.9497 to 1.014
Length of Stay	CR	news2	-0.0198	0.7127	0.9804	0.8789 to 1.087
Length of Stay	CR	GEN HR[1] : Treatment[2]	-0.151	0.7776	0.8598	0.3095 to 2.581
Length of Stay	CR	TZP HR[1] : Treatment[1]	-24.4	0.9999	0.02435	???
Length of Fever	CR	pretreatment[1]	0.302	0.5351	1.353	0.5123 to 3.537
Length of Fever	CR	sex[F]	0.2636	0.5801	1.302	0.5208 to 3.462
Length of Fever	CR	cci	-0.05535	0.6163	0.9462	0.7487 to 1.158
Length of Fever	CR	age	0.005131	0.7904	1.005	0.9698 to 1.047

Length of Fever	CR	news2	0.06695	0.2409	1.069	0.9519 to 1.193
Length of Fever	CR	GEN HR[1] : Treatment[2]	-24.05	0.1254	0.642	???
Length of Fever	CR	TZP HR[1] : Treatment[1]	0.2832	0.4581	0.681	0.4959 to 4.036

Figure S1. Illustration of GEN BCHR status of strains (1= positive, 0=negative) in relation to their MIC concentrations. No association between the isolates MIC and its HR status was found (Point Biserial correlation, p=0.45)

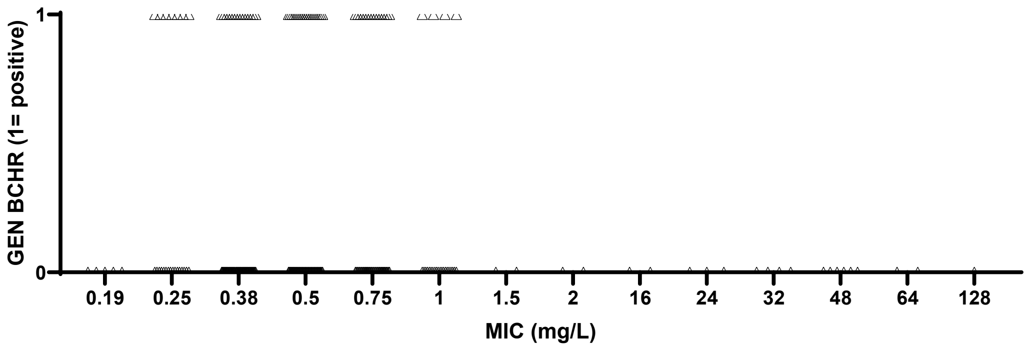

Figure S2. Illustration of TZP BCHR status of strains (1= positive, 0=negative) in relation to their MIC concentrations. A weak correlation (r=0.12, p=0.04 Point Biserial correlation) was found between an isolates MIC and its HR status.

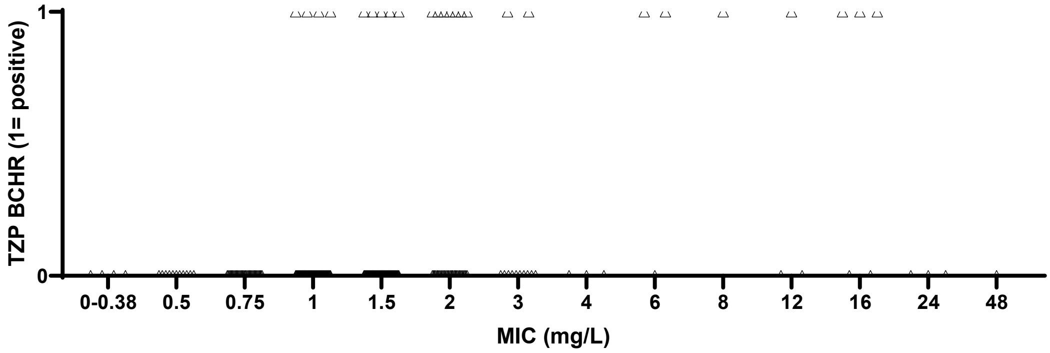

Supplement: 1 [file NIHMS2070931-supplement-1.pdf]
